# MiRSEA: Discovering the pathways regulated by dysfunctional MicroRNAs

**DOI:** 10.18632/oncotarget.10839

**Published:** 2016-07-26

**Authors:** Junwei Han, Siyao Liu, Yunpeng Zhang, Yanjun Xu, Ying Jiang, Chunlong Zhang, Chunquan Li, Xia Li

**Affiliations:** ^1^ College of Bioinformatics Science and Technology, Harbin Medical University, Harbin, 150081, PR China; ^2^ School of Medical Informatics, Daqing Campus, Harbin Medical University, Harbin, 150081, PR China; ^3^ College of Basic Medical Science, Heilongjiang University of Chinese Medicine, Harbin 150040, PR China

**Keywords:** microRNA, mRNA, pathway, cancer, enrichment analysis

## Abstract

Recent studies have shown that dysfunctional microRNAs (miRNAs) are involved in the progression of various cancers. Dysfunctional miRNAs may jointly regulate their target genes and further alter the activities of canonical biological pathways. Identification of the pathways regulated by a group of dysfunctional miRNAs could help uncover the pathogenic mechanisms of cancer and facilitate development of new drug targets. Current miRNA-pathway analyses mainly use differentially-expressed miRNAs to predict the shared pathways on which they act. However, these methods fail to consider the level of differential expression level, which could improve our understanding of miRNA function. We propose a novel computational method, MicroRNA Set Enrichment Analysis (MiRSEA), to identify the pathways regulated by dysfunctional miRNAs. MiRSEA integrates the differential expression levels of miRNAs with the strength of miRNA pathway associations to perform direct enrichment analysis using miRNA expression data. We describe the MiRSEA methodology and illustrate its effectiveness through analysis of data from hepatocellular cancer, gastric cancer and lung cancer. With these analyses, we show that MiRSEA can successfully detect latent biological pathways regulated by dysfunctional miRNAs. We have implemented MiRSEA as a freely available R-based package on CRAN (https://cran.r-project.org/web/packages/MiRSEA/).

## INTRODUCTION

MicroRNAs (miRNAs) are small non-coding RNA molecules that are correlated with regulation of cell homeostasis and various biological processes such as DNA replication, cell development, cell cycle and cell apoptosis [1]. Recent studies have shown that dysfunctional miRNAs are involved in the initiation and progression of various complex diseases, especially cancers, and these dysfunctional miRNAs always participate in regulating several biological pathways [1, 2]. Thus, identifying biological pathways regulated by dysfunctional miRNAs could help us understand disease classification, diagnosis and prognosis [3, 4]. To discover the pathways regulated by a group of dysfunctional miRNAs, the most widely used method compares differentially-expressed miRNAs between states of health and disease, and maps their target genes to biological pathways for enrichment analysis [5]. However, it has been demonstrated that this method usually identifies similar biological pathways even if the phenotypes of interest are very different [6, 7]. Thus, this method may be biased and lead to inaccurate results.

To more accurately identify pathways regulated by dysfunctional miRNAs, several studies integrated matched miRNA and gene expression data. Nam et al. developed a method, MicroRNA and MRNA Integrated Analysis (MMIA), which integrates miRNA and mRNA expression data with miRNA target information to identify the pathways regulated by dysfunctional miRNA sets [8]. MMIA identifies significantly dysfunctional miRNAs from miRNA expression data as well as significantly inversely correlated mRNAs of dysfunctional miRNAs from mRNA expression data. Pathway enrichment analysis is performed for the intersection of predicted target mRNAs and the inversely correlated mRNAs. Xin et al. applied a similar strategy in their work and identified biological pathways regulated by down-regulated and up-regulated miRNAs in MCF7-FR cells [9]. Nevertheless, these methods require matched miRNA and gene expression data sets, which limit their application. Using only miRNA expression profiles to assess the role of miRNAs in regulating biological pathways would greatly expand the utility of the analysis.

Godard et al. proposed an improved method to identify the pathways regulated by dysfunctional miRNAs through enrichment analysis of converted miRNA pathways [7]. In this method, they mapped miRNAs to their pathways according to miRNA-target interactions, and thus pathways of protein-coding genes were converted into lists of miRNAs. A hypergeometric test was then performed by comparing the lists of differentially-expressed miRNAs to the lists of miRNAs in the converted pathways. This method obtains results specific to miRNA signatures, but it treats all differentially-expressed miRNAs equally and fails to consider the effect of miRNA differential expression levels. Similar to genes, prioritizing miRNAs according to their differential expression levels may provide more detailed and comprehensive biological insights. However, this method maps miRNAs to a pathway if only one of their target genes is located in the pathway, and does not consider the total number of genes in the pathways they target.

In the current study, we developed a novel method, MicroRNA Set Enrichment Analysis (MiRSEA), to identify the pathways regulated by a group of dysfunctional miRNAs. MiRSEA integrates the differential expression levels of miRNAs with the strength of miRNA pathway associations, then performs direct enrichment analysis using miRNA expression data. We initially used the hypergeometric test to calculate the weights of miRNAs associated with pathways using miRNA-target interaction information from four public databases (miRTarBase [10], TarBase [11], miRecords [12] and mir2Disease [13]). In this way, pathways of protein-coding genes were converted into pathways of miRNAs according to miRNA-pathway weights. We then evaluated the differential expression levels of miRNAs between two phenotypes of interest. For each converted miRNA pathway, we integrated the differential expression levels of miRNAs and miRNA-pathway weights, and used the weighted Kolmogorov–Smirnov statistic to calculate a pathway enrichment score. Finally, the permutation test was implemented to estimate the statistical significance of enrichment scores. We applied the MiRSEA method to hepatocellular cancer, gastric cancer and lung cancer datasets, and compared our results with two other miRNA-pathway analysis methods. Based on these analyses, we validated that MiRSEA is able to identify the biological pathways regulated by dysfunctional miRNAs in the development of diseases.

## RESULTS

**Figure 1:**
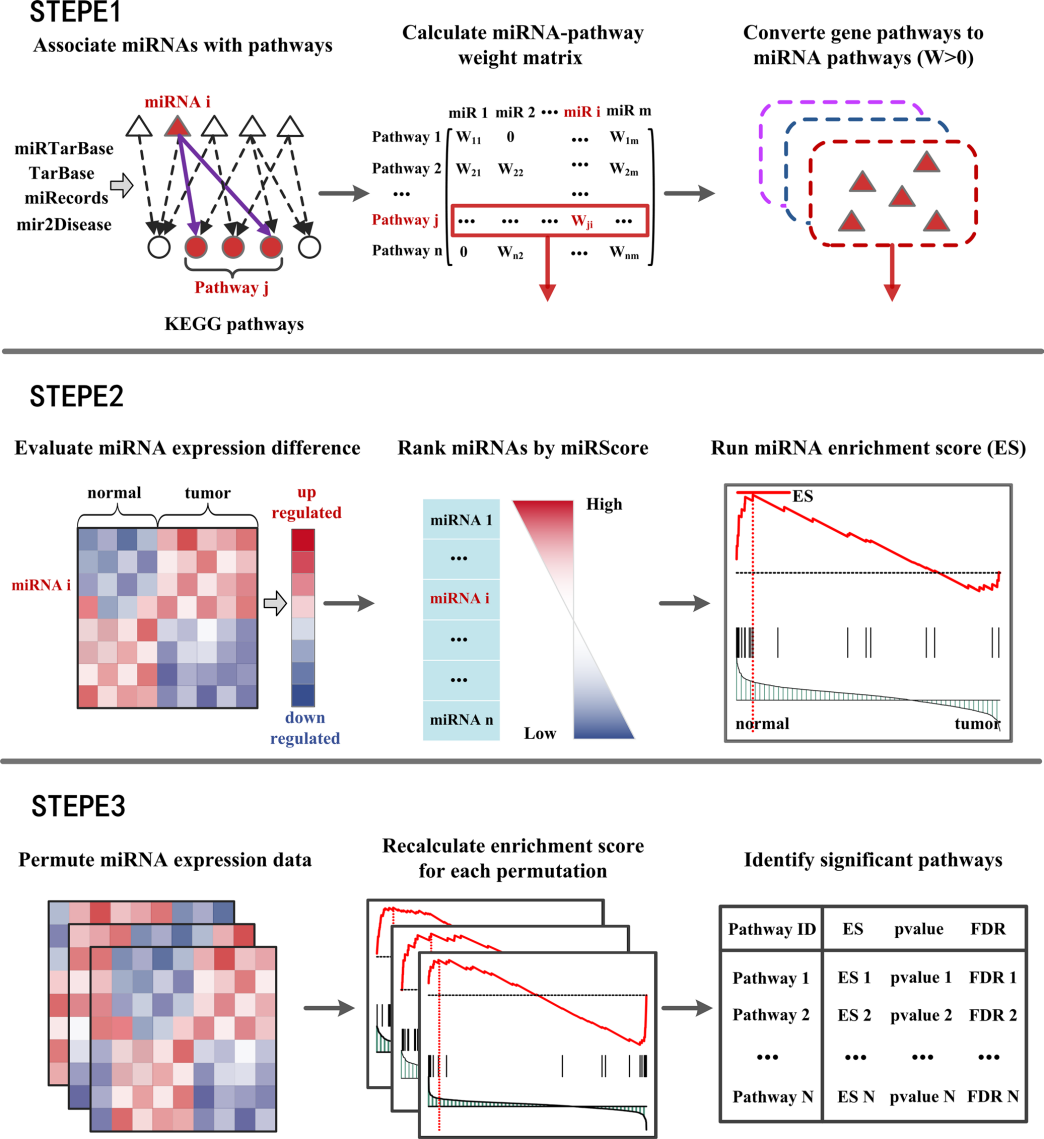
Flow diagram of MiRSEA methodology (**STEPE 1**) miRNAs are associated with KEGG pathways according to four miRNA-target interactions databases. A miRNA-pathway weight matrix is calculated. Pathways of protein-coding genes are converted into pathways of miRNAs with miRNA-pathway weights larger than zero. (**STEPE 2**) For each pathway, the differential expression level of miRNAs and miRNA-pathway weights are integrated into a vector miRScore, and a ranked miRNA list is formed based on the miRScore. MiRNAs in the converted pathway are mapped to the ranked miRNA list, and the miRNA enrichment score of the pathway is calculated by walking down the list. (**STEPE 3**) A permutation test is performed on the miRNA expression data, and pathways are prioritized by FDR after permutation tests.

MiRSEA was developed to identify the pathways regulated by dysfunctional miRNAs through direct enrichment analysis of miRNAs in converted pathways. A workflow diagram of the MiRSEA methodology is shown in Figure 1. In the study, we first demonstrated the ability of MiRSEA to provide biologically meaningful insights using three real miRNA expression datasets from hepatocellular cancer, gastric cancer and lung cancer. In each case, we searched for significant biological pathways regulated by a dysfunctional miRNA set. We then tested if MiRSEA could consistently obtain these results by applying MiRSEA to two independent lung cancer datasets. Finally, we compared the results of MiRSEA with two other pathway enrichment analyses of miRNAs.

### Analyses of hepatocellular carcinoma data

The first case we chose to evaluate the effectiveness of MiRSEA was human hepatocellular carcinoma (HCC) data published by Shih et al. [14]. This dataset contains miRNA expression profiles of 21 non-tumor liver tissues and 68 HCC samples. We applied MiRSEA to identify Kyoto Encyclopedia of Genes and Genomes (KEGG) pathways regulated by dysfunctional miRNAs in the HCC data. All the pathways were prioritized using FDR (Supplementary Table S1).

**Figure 2:**
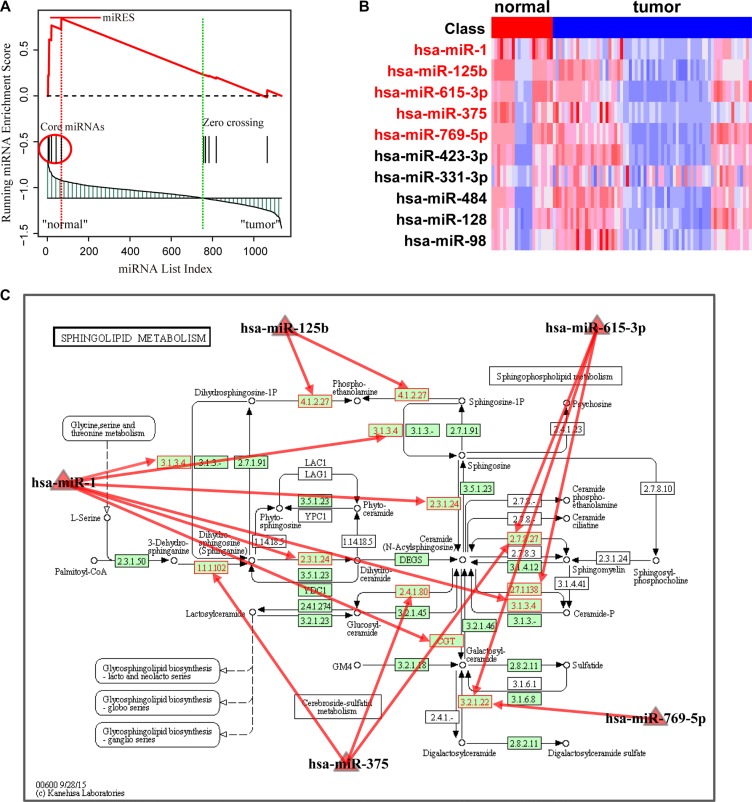
Running enrichment scores and annotating target genes of core miRNAs in the sphingolipid metabolism pathway (**A**) Running-sum statistic is calculated by walking down the miRNA list, and the maximum deviation from zero of the statistic is used as the miRNA enrichment score (miRES) of the pathway. (**B**) Heatmap of the expression levels of miRNAs in the pathway. Core miRNAs are marked in red. (**C**) Sphingolipid metabolism pathway in KEGG. Core miRNAs are mapped to the pathway and the target genes of core miRNAs are annotated in red.

Using FDR < 0.01 pathway significance threshold, the Normal > HCC analysis identified 22 pathways enriched in down-regulated miRNAs in the HCC samples (Table 1). Most of the pathways were readily interpreted in terms of current knowledge of HCC. The most significant pathway was the sphingolipid metabolism pathway, which was proposed to be correlated with tumor cell migration and invasion in hepatocellular carcinoma [15]. We took this pathway as an example to interpret the rationale of the MiRSEA method. Specifically, we first converted pathways of protein-coding genes into pathways of miRNAs with weights, and the weight reflects the strength to which miRNAs regulate pathways. We then defined a *miRScore* to integrate the differential expression level of the miRNA and the miRNA-pathway weight. A ranked miRNA list was constructed according to decreasing *miRScore*. The miRNAs in the converted pathway were mapped to the ranked miRNA list, and a running-sum statistic was calculated by increasing the statistic when we encountered a miRNA in the converted pathway and decreasing it when we encountered miRNAs not in the converted pathway (Figure 2A). The maximum deviation from zero of the statistic was used as the miRNA enrichment score (*miRES*) of the pathway. In a converted miRNA pathway, only a subset of miRNAs in the pathway contributes to the *miRES*, and these miRNAs will typically participate in a biological process. We thus defined the core miRNAs in a pathway to be those miRNAs appearing in the ranked miRNA list *L* at and before the point where *miRES(P)* is obtained (or after if *miRES(P)* < 0). Five core miRNAs were identified in HCC data (Supplementary Table S2). They are miR-1, miR-125b, hsa-miR-615-3p, miR-375 and miR-769-5p. Compared to normal samples, we found these miRNAs were down-regulated in HCC (Figure 2B). Interestingly, reduced expression of these miRNAs, in particular, miR-1, miR-125b and miR-375, has been proposed to be correlated with growth of HCC cells [16–18]. The down-regulation of these miRNAs may result in up-regulation of their targets. By mapping the targets of these miRNAs to the original sphingolipid metabolism pathway, we found that a high number of genes in the pathway were targeted (Figure 2C). This may alter the properties of sphingolipid metabolism in HCC and contribute to cancer progression [19].

**Table 1:** Pathways identified by MiRSEA with FDR < 0.01 in the HCC dataset

Pathway	Size of miRNA^a^	NmiRES	FDR	Character
Sphingolipid metabolism	10	2.17	< 0.001	Down-regulated
Calcium signaling pathway	45	2.02	< 0.001	Down-regulated
Cell adhesion molecules (CAMs)	32	2.01	< 0.001	Down-regulated
Glycerolipid metabolism	13	1.94	< 0.001	Down-regulated
Non-homologous end-joining	12	1.92	< 0.001	Down-regulated
Drug metabolism - other enzymes	11	1.90	< 0.001	Down-regulated
Prion diseases	27	1.90	< 0.001	Down-regulated
Cysteine and methionine metabolism	25	1.89	< 0.001	Down-regulated
Focal adhesion	124	1.83	< 0.001	Down-regulated
Adherens junction	78	1.83	< 0.001	Down-regulated
Pyruvate metabolism	17	1.78	< 0.001	Down-regulated
MAPK signaling pathway	106	1.75	< 0.001	Down-regulated
Bladder cancer	100	1.71	< 0.001	Down-regulated
Pathways in cancer	177	1.64	< 0.001	Down-regulated
Starch and sucrose metabolism	17	1.79	0.005	Down-regulated
Type II diabetes mellitus	37	1.76	0.005	Down-regulated
Glioma	103	1.70	0.005	Down-regulated
Endometrial cancer	90	1.66	0.005	Down-regulated
p53 signaling pathway	102	1.60	0.005	Down-regulated
Prostate cancer	133	1.57	0.005	Down-regulated
Viral myocarditis	56	1.78	0.009	Down-regulated
Colorectal cancer	96	1.56	0.009	Down-regulated
Ribosome	37	−1.91	< 0.001	Up-regulated
Chemokine signaling pathway	77	−1.90	< 0.001	Up-regulated
Huntingtons disease	59	−1.83	< 0.001	Up-regulated
Tight junction	76	−1.69	< 0.001	Up-regulated
Melanogenesis	49	−1.65	< 0.001	Up-regulated
Phosphatidylinositol signaling system	37	−1.61	< 0.001	Up-regulated
Leishmania infection	36	−1.71	0.0059	Up-regulated
Renal cell carcinoma	78	−1.69	0.0059	Up-regulated
Antigen processing and presentation	31	−1.80	0.009	Up-regulated
Fructose and mannose metabolism	21	−1.73	0.009	Up-regulated
Pentose phosphate pathway	22	−1.64	0.009	Up-regulated

a the number of miRNAs in the converted pathway.

The complementary analysis (HCC>Normal) identified 11 statistically significant pathways enriched by up-regulated miRNAs in HCC with FDR<0.01 (Table 1). Almost all are clearly demonstrated to be associated with HCC. These pathways include the chemokine signaling pathway, which plays a paramount role in HCC progression, growth and metastasis and immune response to HCC [20, 21]; genes encoding proteins in the tight junction pathway that are associated with recurrence of primary HCC [22]; and kinases in the phosphatidylinositol signaling pathway that mediate acquired resistance to sorafenib (a drug approved by the United States Food and Drug Administration for HCC) in HCC cells [23]. Specifically, in the chemokine signaling pathway, 25 core miRNAs were identified (Supplementary Figure S1A and Supplementary Table S3) and their expression was up-regulated in HCC (Supplementary Figure S1B). They include miR-96, miR-221, miR-182 and miR-135a, which may be associated with initiation and progression of HCC. For instance, miR-96 was frequently found to be up-regulated in HCC [24]. Overexpression of miR-221 stimulated growth of tumorigenic murine hepatic progenitor cells, and the use of synthetic inhibitors of miR-221 was shown to be a promising approach in liver cancer treatment [25]. Wang et al. found that miR-182 was up-regulated in a rat model of HCC and could act as an HCC oncogene [26]. Moreover, miR-135a promoted invasion and metastasis *in vitro* and in mouse models of HCC [27]. The accumulation of these core miRNAs may jointly alter the activity of chemokine signaling pathway in HCC.

### Analyses of gastric adenocarcinoma data

The second case we chose to evaluate was miRNA expression data of gastric adenocarcinoma (GC) [28]. This dataset compiles the expression of miRNAs from 60 primary GC tissues and eight surrounding non-cancerous tissues. We applied MiRSEA to this dataset, with the full list of ranked pathways shown in Supplementary Table S4.

**Figure 3:**
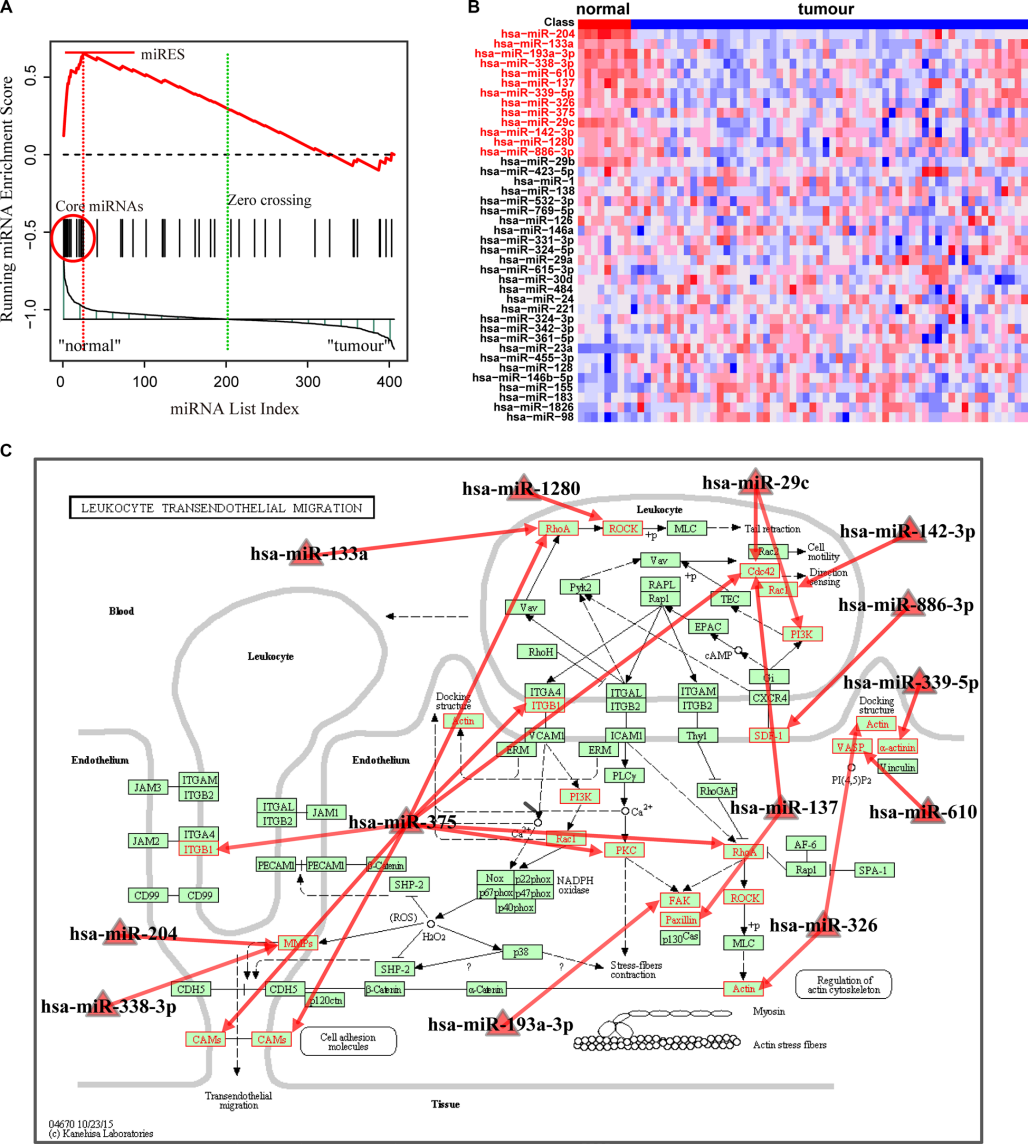
Running enrichment scores and annotating target genes of core miRNAs in the leukocyte transendothelial migration pathway (**A**) Running-sum statistic is calculated by walking down the miRNA list, and the maximum deviation from zero of the statistic is used as the miRNA enrichment score (miRES) of the pathway. (**B**) Heatmap of the miRNAs in the pathway. Core miRNAs are marked in red. (**C**) Leukocyte transendothelial migration pathway in KEGG. Core miRNAs are mapped to the pathway and the target genes of core miRNAs are annotated in red.

With FDR < 0.01, the Normal > GC analysis identified 15 significant pathways enriched by down-regulated miRNAs in the GC samples (Table 2). These include pathways involving leukocyte transendothelial migration, focal adhesion, Fc gamma R-mediated phagocytosis and selenoamino acids metabolism. These pathways were enriched with miRNAs associated with normal tissue function, and their dysfunction may promote the generation and development of GC. For instance, the most significant pathway involved leukocyte transendothelial migration, which was reported to be a critical component in tumor progression [29]. For this pathway, the miRNA enrichment score was obtained by calculation of the running-sum statistic (Figure 3A), and 13 core miRNAs were identified (Supplementary Table S5). The core miRNAs, including miR-204, miR-133a and miR-610, were down-regulated in GC samples (Figure 3B) and are clearly correlated with gastric cancer. Sacconi et al. showed that down-regulation of miR-204 promoted colony-forming ability, migration and tumor engraftment of GC cells [30]. And miR-133a was proposed to be correlated with the proliferation, invasion and cell cycle progression of GC cells [31]. Finally, Wang et al. found that miR-610 functioned as a tumor suppressor miRNA and was down-regulated in GC, which may represent a novel therapeutic approach to limit GC metastasis [32]. These core miRNAs may modulate their pathways through their targets in the pathway. We further mapped the targets of these core miRNAs to the original pathway and found a number of genes were targeted (Figure 3C). Interestingly, overexpression of these targets, such as matrix metalloproteinase-9, ras homolog family member A and vasodilator-stimulated phosphoprotein, have been demonstrated to be associated with tumor migration and invasion [32–34]. The second significant pathway was focal adhesion, in which kinase gene amplification was significantly correlated with cancer progression and poor prognosis in GC [35]. In this pathway, 21 core miRNAs were identified (Supplementary Table S6). These dysfunctional miRNAs may cooperatively change the activity of the original pathway by regulating their targets.

**Table 2:** Pathways identified by MiRSEA with FDR < 0.01 in the gastric adenocarcinoma dataset

Pathway	Size of miRNA^a^	NmiRES	FDR	Character
Leukocyte transendothelial migration	40	2.24	< 0.001	Down-regulated
Focal adhesion	79	2.04	< 0.001	Down-regulated
Selenoamino acids metabolism	10	2.03	< 0.001	Down-regulated
Fc gamma R-mediated phagocytosis	40	1.97	< 0.001	Down-regulated
Pathways in cancer	111	1.87	< 0.001	Down-regulated
VEGF signaling pathway	48	1.827	< 0.001	Down-regulated
Small cell lung cancer	73	1.79	< 0.001	Down-regulated
Regulation of actin cytoskeleton	59	1.77	< 0.001	Down-regulated
Colorectal cancer	70	1.68	< 0.001	Down-regulated
Notch signaling pathway	23	1.78	0.009	Down-regulated
Chemokine signaling pathway	59	1.78	0.009	Down-regulated
Cysteine and methionine metabolism	23	1.76	0.009	Down-regulated
Leishmania Infection	29	1.72	0.009	Down-regulated
MAPK signaling pathway	74	1.60	0.009	Down-regulated
Prostate cancer	86	1.55	0.009	Down-regulated
One carbon pool by folate	12	−1.72	0.014	Up-regulated
Amino sugar and nucleotide sugar metabolism	15	−1.53	0.055	Up-regulated

a the number of miRNAs in the converted pathway.

The reciprocal analysis (GC > Normal) did not yield any significant pathway with FDR<0.01; however, two pathways fell just short of the significance threshold (FDR < 0.01) (Table 2). These two pathways are the one carbon pool by folate pathway correlated with the chemoprevention of gastric carcinogenesis [36], and the amino sugar and nucleotide sugar metabolism pathway which may be related to gastric cancer risk [37]. As these pathways were enriched with up-regulated miRNAs in the GC samples, their activity may be altered by the miRNAs in the development of GC.

### Analyses of lung cancer data

To further evaluate the results of MiRSEA, we utilized sample-matched miRNA-mRNA expression datasets of non-small cell lung cancer (NSCLC; GSE29248 and GSE29249) [49], which included six NSCLC tissues and six adjacent normal tissues. We first applied MiRSEA to the miRNA expression data and then validated the results using the sample-matched mRNA expression data. With FDR < 0.01, MiRSEA found 64 significant pathways enriched by up-regulated miRNAs in NSCLC. These pathways, such as the gonadotropin-releasing hormone (GnRH) signaling, Fc gamma R-mediated phagocytosis and gap junction pathway, have been well reported to be associated with the initiation and development of NSCLC in the published literatures [38–40]. To verify if these pathways were modulated by dysregulated miRNAs, we examined changes in the expression of genes targeted by dysregulated miRNAs in the pathways. For the GnRH signaling pathway, we identified 22 core miRNAs that were up-regulated in NSCLC (Supplementary Figure S2A). By intersecting the targets of core miRNAs and genes in the pathway, we obtained 25 genes. Interestingly, 72% (18/25) of the intersecting genes were down-regulated in the NSCLC samples (Supplementary Figure S2B). In the same way, we found that 62% (18/29) and 52% (11/21) of the intersecting genes were down-regulated in the Fc gamma R-mediated phagocytosis and gap junction pathways, respectively (Supplementary Figures S3 and S4). These results indicate that the pathways identified by MiRSEA may be controlled by dysregulated miRNAs in NSCLC.

**Figure 4:**
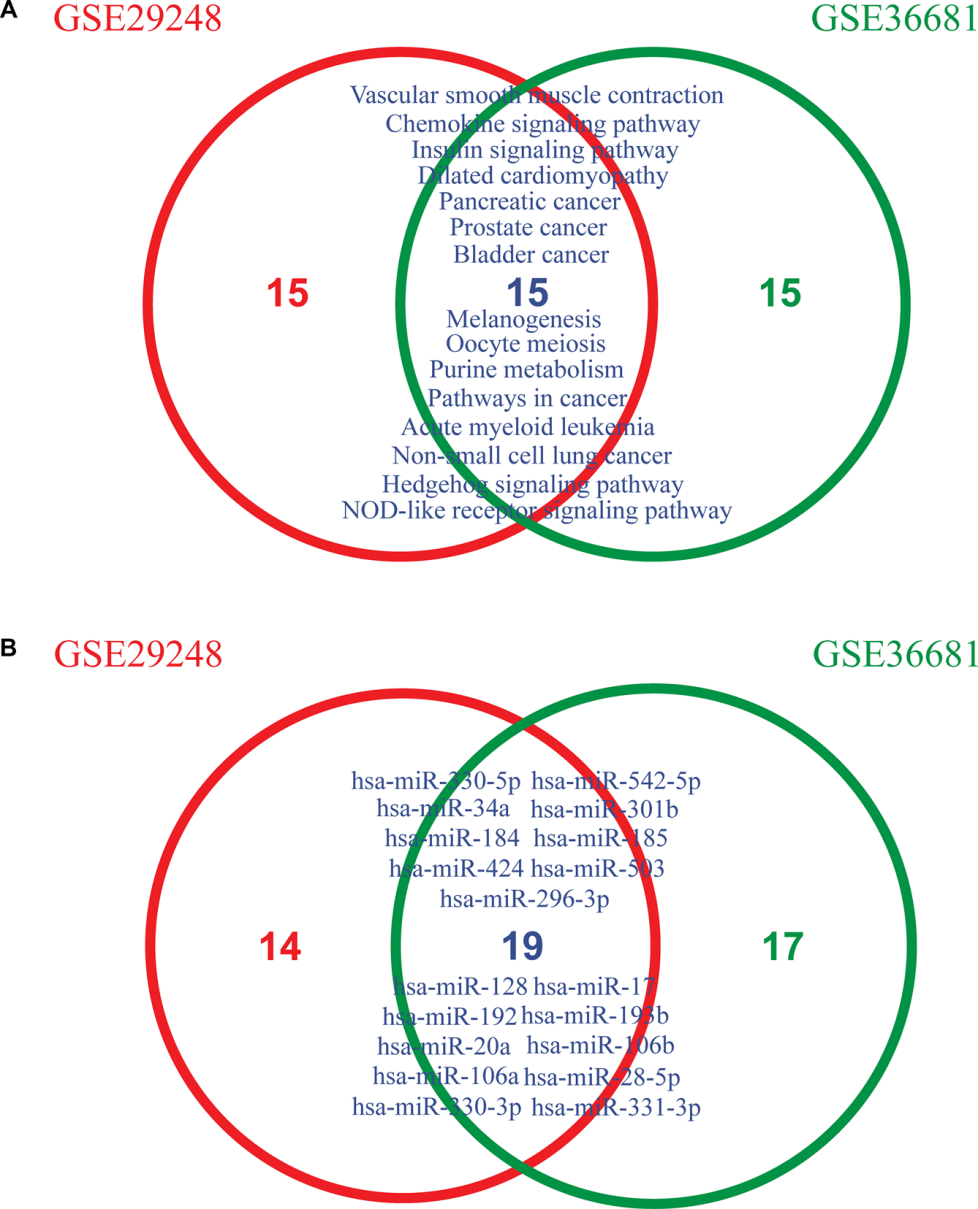
(**A**) Overlapping pathways between two lung cancer datasets (GSE29248 and GSE36681) across the top 30 pathways. (**B**) Overlapping core miRNAs of the non-small cell lung cancer pathway from GSE29248 and GSE36681 datasets, respectively.

To determine if MiRSEA could obtain consistent results, we applied MiRSEA to another NSCLC dataset (GSE36681). With FDR<0.01, 56 significant pathways were identified. We compared the results from GSE29248 and GSE36681 datasets. To provide a more general comparison, we used the top 30 pathways from each dataset to test how many pathways are overlapped. The results showed that approximately 50% (14/30) pathways were shared between the two datasets (Figure 4A). These pathways include the insulin signaling pathway, hedgehog signaling pathway and focal adhesion pathway, which have been demonstrated to be correlated with NSCLC in published literature [41–43]. Furthermore, the NSCLC pathway was also identified as overlapped between the two datasets. We further tested if this pathway identified from different datasets was regulated by the same miRNAs. Interestingly, 33 and 36 core miRNAs were identified in the NSCLC pathway from the GSE29248 and GSE36681 datasets, respectively, of which 18 miRNAs were shared (Figure 4B). The shared miRNAs include miR-503, which is correlated with the development of drug resistance in human NSCLC [44]; miR-34a, which mediates suppression of Notch-1 expression in NSCLC cell lines [45]; miR-128, shown to be correlated with human NSCLC tumorigenesis, angiogenesis and lymphangiogenesis [46]; and miR-106a and miR-192, which are both overexpressed in NSCLC [47]. These core miRNAs may contribute to altering the properties of the NSCLC pathway. The above analyses thus indicate that the MiRSEA method is able to provide consistent results from different datasets with the same phenotype.

### Comparison of MiRSEA with two other methods

To further explore whether MiRSEA could provide new biological insights, we compared the results of MiRSEA with two other miRNA pathway analyses. The first was enrichment analysis of miRNA target genes, which has been widely used in recent studies (we here refer to it as the Traditional method [5]). This method identifies the original differentially-expressed miRNAs and then maps their target genes to the pathways for enrichment analysis. The second method was enrichment analysis of miRNAs proposed by Godard et al. [7]. In this method, miRNAs are mapped to a pathway when at least one of their target genes is located in that pathway. The enrichment analysis is conducted by comparing differentially-expressed miRNAs to the lists of miRNAs in different pathways. The differential expression analysis was performed by *Student t-test* and the miRNAs with FDR < 0.01 were deemed as differentially expressed.

We applied MiRSEA, the Traditional method and the Godard et al. method to identify KEGG pathways regulated by dysfunctional miRNAs in five cancer datasets, including human HCC (GSE36915), gastric adenocarcinoma (GSE26595), oral carcinoma (GSE45238), acute lymphoblastic leukemia (GSE56489) and lung cancer (GSE36681). The number of pathways detected by each method for all datasets is provided in Supplementary Table S7. For the human HCC data, MiRSEA identified 33 significant pathways (22 HCC-downregulated and11 HCC-upregulated pathways) with a pathway significant threshold of FDR<0.01. In contrast, the Traditional method found 60 significant pathways (33 HCC-downregulated and 27 HCC-upregulated pathways) and the Godard et al. method did not identify any statistically significant pathways in the HCC dataset. By comparing the results, 21 pathways identified by MiRSEA were missed by the other two methods (Supplementary Table S8). Interestingly, these pathways, such as the sphingolipid metabolism pathway [15], the phosphatidylinositol signaling system [23], cysteine and methionine metabolism pathways [48] and pyruvate metabolism [49], have been thoroughly documented as related to HCC. These results indicate that MiRSEA may be able to identify new pathways regulated by dysfunctional miRNAs in the development of HCC. Moreover, the MiRSEA method can identify the core miRNAs for significant pathways which may alter the activity of those pathways. Although the Traditional method also identified several pathways exclusively, it was found that even random and meaningless miRNA lists produced significant functional pathways using this method [6, 7]. Thus, the Traditional method is biased and leads to inaccurate results. Additionally, the Godard et al. method did not yield any statistically significant pathways. This may have occurred because the Godard et al. method uses only differentially-expressed miRNAs to identify pathways and ignores the effect of several modestly cancer-related miRNAs. Moreover, the extent to which miRNAs are associated with pathways is ignored by this method. In fact, the MiRSEA method, which integrates the differential expression level of miRNAs and the extent of miRNA- associated pathways, may complement the Godard et al. method. In agreement with these results, MiRSEA also identified three pathways in GC data, zero pathways in oral carcinoma data, 40 pathways in acute lymphoblastic leukemia data and nine pathways in lung cancer data, which were missed by the other two methods. These results indicate that MiRSEA may detect new biological pathways regulated by dysfunctional miRNAs.

## DISCUSSION

MiRNAs are correlated with regulating DNA replication, cell development, cell cycle and cell apoptosis by inhibiting the expression of their target genes. Dysfunctional miRNAs may promote the occurrence and development of various cancers. In systems biology, dysfunctional miRNAs may jointly activate or inhibit their target genes and further alter the activity of their canonical biological pathways. Thus, elucidating the pathways regulated by a group of dysfunctional miRNAs may help uncover the pathogenic mechanisms of diseases and identify new drug targets. Here, MiRSEA was developed to identify canonical pathways regulated by dysfunctional miRNA sets. MiRSEA applies the degree to which miRNAs are differentially expressed which has been neglected by recent miRNA-pathway analyses. Moreover, considering that a miRNA may be more likely to regulate a pathway as the number of genes it targets in the pathway increases, we used the hypergeometric test to calculate the weight for each pair of miRNAs and pathways containing the miRNA-target interaction (See Methods). However, any other suitable metric, such as Bleazard's unbiased analysis [6], can be used to calculate this weight. To avoid false positive interactions from miRNA-target prediction algorithms, we collected experimentally validated miRNA-target interaction data from four public databases (miRTarBase (V4.5), TarBase (V6.0), miRecords (V4.0) and mir2Disease). Using the miRNA-pathway weights, we converted pathways of protein-coding genes into pathways composed of miRNAs. Finally, we mapped the miRNA expression data to the converted miRNA pathways, and used the weighted Kolmogorov–Smirnov statistic to perform direct enrichment analysis.

We applied the MiRSEA method to HCC, GC and two NSCLC datasets. Based on these analyses, we demonstrated that MiRSEA can effectively identify pathways enriched by dysfunctional miRNAs. Moreover, for each significant pathway, MiRSEA can provide the core miRNAs which may alter pathway activity. For the gastric adenocarcinoma data, the most significant pathway was that for leukocyte transendothelial migration, in which we identified 13 core miRNAs (Supplementary Table S5). Most of these core miRNAs, including miR-204, miR-133a and miR-610, have been clearly demonstrated to be associated with gastric cancer in the published literature [30–32]. Although the roles of some core miRNAs are unknown in gastric cancer, their identification may help biologists understand the pathogenic mechanism of cancer and develop new therapeutic targets.

We also compared the results of MiRSEA with two other miRNA pathway analyses, namely, what we call the Traditional method [5] and the method of Godard et al. [7], using five cancer datasets (Supplementary Table S7). For instance, in the HCC dataset, MiRSEA identified 33 significant pathways (22 HCC-downregulated and 11 HCC-upregulated pathways) with FDR < 0.01, while the Traditional method identified 60 significant pathways (33 HCC-downregulated and 27 HCC-upregulated pathways). By comparing these results, we found 21 pathways which were identified by MiRSEA as highly related to HCC that were not detected by the Traditional method, including pathways involving sphingolipid metabolism [15], the phosphatidylinositol signaling system [23], cysteine and methionine metabolism [48] and pyruvate metabolism [49]. Although the Traditional method also identified several pathways exclusively, it has been shown to produce similar biological pathways with even random and meaningless miRNA sets [6, 7]. Thus, the Traditional method is apparently biased and may lead to inaccurate results. In addition, the Godard et al. method yielded zero significant pathways. This may have occurred because this method only compares the lists of differentially-expressed miRNAs to the lists of miRNAs in the different converted pathways, which may ignore the effect of several modestly cancer-related miRNAs. Moreover, the Godard et al. method performs enrichment analysis directly based on miRNAs, regardless of the number of genes in the pathway they targeted. MiRSEA, which integrates the weight of miRNA-pathway association and miRNA differential expression level, can effectively identify the pathways regulated by dysfunctional miRNAs in the disease phenotype.

Some studies have integrated miRNA and gene expression data to discover pathways regulated by dysfunctional miRNAs [8, 9]. Although these studies introduced more information for analysis and produced several significant results, they still required both miRNA and gene expression data, which may limit their application. Thus, there remains an unfulfilled need for more powerful and accurate tools to identify biological pathways regulated by dysfunctional miRNAs using only miRNA expression data.

To make MiRSEA broadly applicable, MiRSEA was implemented as a freely available R-based package on CRAN (https://cran.r-project.org/web/packages/MiRSEA/). The user only needs to input miRNA expression data from normal and disease samples, and the MiRSEA package will produce a prioritized list of pathways regulated by dysfunctional miRNAs. MiRSEA was applied to KEGG pathways in this study, but it can also be applied to other pathway databases and functional biological categories.

## MATERIALS AND METHODS

### Materials

Experimentally-validated miRNA-target interactions were used in the study. We collected the miRNA targets from four public databases (miRTarBase (V4.5) [10], TarBase (V6.0) [11], miRecords (V4.0) [12] and mir2Disease [13]). For humans, 12 113 targets are associated with 576 miRNAs in miRTarBase, 11 933 targets are associated with 217 miRNAs in TarBase, 1105 targets are associated with 244 miRNAs in miRecords and 403 targets are associated with 181 miRNAs in mir2Disease. We merged these databases to obtain 40 990 human-specific miRNA-target interactions including 15 917 genes and 879 miRNAs. Pathways of protein coding genes were obtained from the KEGG database [50]. Additionally, we downloaded 186 KEGG pathways from the Molecular Signatures Database (MSigDB, http://www.broadinstitute.org/gsea/index.jsp) [51].

To illustrate the effectiveness of MiRSEA, we used three cases of miRNA expression profiles from the Gene Expression Omnibus database (http://www.ncbi.nlm.nih.gov/geo/). The first case was a human hepatocellular carcinoma (HCC) analysis published by Shih et al. (accession no. GSE36915) [14]. This data contains miRNA expression profiles of 68 HCC and 21 non-tumor liver tissues. The second case, obtained from Lim et al., was an analysis of miRNA expression in primary gastric adenocarcinoma (GC; accession no. GSE26595) [28] and includes data from 60 primary GC tissues and eight surrounding non-cancerous tissues. The third case utilized two independent non-small cell lung cancer (NSCLC) datasets (accession no. GSE29248 [52] and GSE36681 [53]). One dataset analyzed six NSCLC cancer tissues and six adjacent normal tissues, and the other examined 56 NSCLC cancer tissues and 56 uninvolved lungs.

### Converting pathways of protein-coding genes into miRNA pathways

To perform direct enrichment analysis of miRNAs, we converted pathways of protein-coding genes into pathways of miRNAs. A miRNA primarily regulates biological pathways through its targets. We defined a weight of miRNAs associated with pathways (miRNA-pathway weight), which was used to reflect the strength to which miRNAs regulate pathways. We used the hypergeometric test to calculate the miRNA-pathway weight for each pair of miRNAs and pathways. The weight of the association between miRNA *i* and pathway *j* (*W_ij_*) is given as follows:

$$W_{\text{ij}}=1-p$$ (1)

$$p=\sum_{x=r}^{n}\frac{\left(\begin{array}{c} t\\ x \end{array}\right)\left(\begin{array}{c} m-t\\ n-x \end{array}\right)}{\left(\begin{array}{c} m\\ n \end{array}\right)}$$ (2)

where *m* represents the number of genes in the whole genome; *t* is the number of genes involved in pathway *j*; *n* represents the number of targets of miRNA *i*; *r* is the number of overlaps between targets of miRNA *i* and genes in pathway *j*. In this way, each miRNA is assigned a weight with pathway *j*. If miRNA *i* does not target any one of the genes in pathway *j*, the *W_ij_* will be zero; otherwise, *W_ij_* will be larger than zero, and a large *W_ij_* indicates that miRNA *i* regulates pathway *j* to a large extent. Thus, each pair of miRNAs and pathways is assigned a weight. Then, we constructed a weighted miRNA-pathway matrix. When *W_ij_* > 0, miRNA *i* was mapped to pathway *j* to form a miRNA pathway. To avoid overly narrow or broad functional categories, pathways with more than 10 miRNAs and less than 200 miRNAs were used for direct enrichment analysis in the following.

### Calculating the enrichment score for each converted pathway

If the miRNAs in a converted pathway possess both large differential expression levels and miRNA-pathway weights, that pathway will be regulated by the miRNAs in a specific phenotype. We thus defined a *miRScore* to integrate the differential expression level of the miRNA and the miRNA-pathway weight. The formula for *miRScore* is given by:

$$\text{miRScore}=\left(1+\text{Wi}\right)\times DE_{i})$$ (3)

where *W_i_* is the weight of miRNA *i* with a given pathway and *DE_i_* is the differential expression level of miRNA *i*. We used the signal-to-noise ratio (*S2N*) [51] to evaluate the differential expression levels of miRNAs in the study. However, any other suitable metric can be used. A miRNA in a pathway with *miRScore* greatly different from zero indicates that the miRNA would probably regulate the pathway in the specific phenotype.

For a given pathway, the weight for each miRNA in an expression profile associated with the pathway was initially calculated. We then ranked the *N* miRNAs in the profile to form a miRNA list *L* = <*miR_1_*, *miR_2_*, …, *miR_N_*> according to decreasing *miRScore*. We mapped the miRNAs involved in the given pathway (miRNAs with *W* > 0) to the ranked miRNA list *L*. Obviously, when the miRNAs in a given pathway cluster at the top or bottom of list *L,* that pathway may be regulated by a group of dysfunctional miRNAs in the specific phenotype. We used a weighted Kolmogorov–Smirnov statistic used previously in the gene set enrichment analysis method [51] to calculate a miRNA enrichment score (*miRES*), which reflects the degree to which the pathway is overrepresented toward the extremes (top or bottom) of the ranked miRNA list *L*. The *miRES* was calculated by walking down the ranked miRNA list *L*, increasing a running-sum statistic when we encountered a miRNA in the pathway and decreasing it when we encountered a miRNAs not in the pathway. In detail, at a given position *k* in list *L*, we evaluated the fraction of miRNAs in the pathway (*F_hit_*) weighted by their *miRScores* and the fraction of miRNAs not in the pathway (*F_miss_*) as follows:

$$F_{\text{hit}}\left(\text{pathway},k\right)=\sum_{\begin{array}{c} {\text{miR}}_{l}\in \text{pathway}\\ l\leq k \end{array}}\frac{{\left|r_{l}\right|}^{q}}{N_{R}},\text{where }N_{R}=\sum_{{\text{miR}}_{l}\in \text{pathway}}{\left|r_{l}\right|}^{q}$$ (4)

$$F_{\text{miss}}\left(\text{pathway},k\right)=\sum_{\begin{array}{c} {\text{miR}}_{l}\notin \text{pathway}\\ l\leq k \end{array}}\frac{1}{N_{\text{miss}}}$$ (5)

where *r_l_* represents the *miRScore* of miRNA *l*, which reflects the extent of differential expression of the miRNA and the miRNA-pathway weight, and *N_miss_* represents the number of miRNAs in the list *L* not in the pathway. With the position *k* walking down the miRNA list *L*, the *miRES* of the pathway (*miRES(P)*) was calculated as the maximum deviation from zero of *F_hit_*−*F_miss_*. If the miRNAs are randomly distributed in the miRNA list, the *miRES(P)* will be relatively small, but if the miRNAs cluster at the top or bottom of the list, the *miRES(P)* will be high. The parameter *q* was used to weight miRNAs in the pathway with their *miRScores* normalized by the sum of the *miRScores* over all for the miRNAs in the pathway. We set *q* = 1 for the examples in this study. For some particular phenotypes, if only a small subset of miRNAs in the pathways are overrepresented toward the extremes in the miRNA list, it may be desirable to set *q* > 1. For each converted miRNA pathway, the respective *miRES* was calculated in the above way.

In a converted miRNA pathway, only a subset of miRNAs in the pathway contributes to the *miRES*, and these miRNAs will contribute to a biological process. Thus, it is meaningful to extract the core miRNAs of the converted miRNA pathways. Here, we defined the core miRNAs in a pathway to be those miRNAs appearing in the ranked miRNA list *L* at and before the point where *miRES(P)* is obtained (or after if *miRES(P)* < 0).

### Estimating the statistical significance of the enrichment score

We performed a phenotype-based permutation test procedure, which preserves the complex correlation structure of miRNA expression, to estimate the statistical significance (empirical *p-* value) of the *miRES* for each pathway. Specifically, we redistributed the phenotype labels of miRNA expression data and recomputed the *miRES* for the permuted data. This generates a background set of *miRES* after performing *N* permutations, which was designated as *miRES_perm_*. The empirical *p-* value was computed as *p*-value = *M/N*, where *M* is the number of *miRES_perm_* greater than the observed *miRES(P)* when the observed *miRES(P)* > 0, or *M* is the number of *miRES_perm_* less than the observed *miRES(P)* when the observed *miRES(P)* < 0;*N* represents the number of permutation times, and was set at 1000 in this study. The phenotype-based permutation preserves miRNA-miRNA correlations and thus would be reasonable for biological assessment of significance. To correct for multiple hypotheses testing, we applied the false discovery rate (FDR) method proposed by Benjamini and Hochberg [54] to adjust the empirical *p-* value. In our study, the FDR at 0.05 was used as the pathway significance threshold.

We further normalized the *miRES* for each converted miRNA pathway to account for the size of the pathway and allow inter-pathway comparisons with *miRES*. The normalized miRNA enrichment scores (*NmiRES*) for the pathways were produced by separately rescaling the observed positive and negative enrichment scores by dividing by the mean of positive and negative scores in the *miRES_perm_*.

## SUPPLEMENTARY MATERIALS













## References

[1] Mo YY (2012). MicroRNA regulatory networks and human disease. Cell Mol Life Sci.

[2] Calin GA, Croce CM (2006). MicroRNA signatures in human cancers. Nature reviews.

[3] Croce CM (2009). Causes and consequences of microRNA dysregulation in cancer. Nat Rev Genet.

[4] Kent OA, Mendell JT (2006). A small piece in the cancer puzzle: microRNAs as tumor suppressors and oncogenes. Oncogene.

[5] Liu B, Li J, Cairns MJ (2014). Identifying miRNAs, targets and functions. Brief Bioinform.

[6] Bleazard T, Lamb JA, Griffiths-Jones S (2015). Bias in microRNA functional enrichment analysis. Bioinformatics (Oxford, England).

[7] Godard P, van Eyll J (2015). Pathway analysis from lists of microRNAs: common pitfalls and alternative strategy. Nucleic Acids Res.

[8] Nam S, Li M, Choi K, Balch C, Kim S, Nephew KP (2009). MicroRNA and mRNA integrated analysis (MMIA): a web tool for examining biological functions of microRNA expression. Nucleic Acids Res.

[9] Xin F, Li M, Balch C, Thomson M, Fan M, Liu Y, Hammond SM, Kim S, Nephew KP (2009). Computational analysis of microRNA profiles and their target genes suggests significant involvement in breast cancer antiestrogen resistance. Bioinformatics (Oxford, England).

[10] Hsu SD, Lin FM, Wu WY, Liang C, Huang WC, Chan WL, Tsai WT, Chen GZ, Lee CJ, Chiu CM, Chien CH, Wu MC, Huang CY (2011). miRTarBase: a database curates experimentally validated microRNA-target interactions. Nucleic Acids Res.

[11] Vergoulis T, Vlachos IS, Alexiou P, Georgakilas G, Maragkakis M, Reczko M, Gerangelos S, Koziris N, Dalamagas T, Hatzigeorgiou AG (2012). TarBase 6. 0: capturing the exponential growth of miRNA targets with experimental support. Nucleic Acids Res.

[12] Xiao F, Zuo Z, Cai G, Kang S, Gao X, Li T (2009). miRecords: an integrated resource for microRNA-target interactions. Nucleic Acids Res.

[13] Jiang Q, Wang Y, Hao Y, Juan L, Teng M, Zhang X, Li M, Wang G, Liu Y (2009). miR2Disease: a manually curated database for microRNA deregulation in human disease. Nucleic Acids Res.

[14] Shih TC, Tien YJ, Wen CJ, Yeh TS, Yu MC, Huang CH, Lee YS, Yen TC, Hsieh SY (2012). MicroRNA-214 downregulation contributes to tumor angiogenesis by inducing secretion of the hepatoma-derived growth factor in human hepatoma. J Hepatol.

[15] Bao M, Chen Z, Xu Y, Zhao Y, Zha R, Huang S, Liu L, Chen T, Li J, Tu H, He X (2012). Sphingosine kinase 1 promotes tumour cell migration and invasion via the S1P/EDG1 axis in hepatocellular carcinoma. Liver Int.

[16] Datta J, Kutay H, Nasser MW, Nuovo GJ, Wang B, Majumder S, Liu CG, Volinia S, Croce CM, Schmittgen TD, Ghoshal K, Jacob ST (2008). Methylation mediated silencing of MicroRNA-1 gene and its role in hepatocellular carcinogenesis. Cancer Res.

[17] Li W, Xie L, He X, Li J, Tu K, Wei L, Wu J, Guo Y, Ma X, Zhang P, Pan Z, Hu X, Zhao Y (2008). Diagnostic and prognostic implications of microRNAs in human hepatocellular carcinoma. Int J Cancer.

[18] He XX, Chang Y, Meng FY, Wang MY, Xie QH, Tang F, Li PY, Song YH, Lin JS (2012). MicroRNA-375 targets AEG-1 in hepatocellular carcinoma and suppresses liver cancer cell growth *in vitro* and *in vivo*. Oncogene.

[19] Ogretmen B, Hannun YA (2004). Biologically active sphingolipids in cancer pathogenesis and treatment. Nature reviews.

[20] Huang F, Geng XP (2010). Chemokines and hepatocellular carcinoma. World J Gastroenterol.

[21] Chew V, Chen J, Lee D, Loh E, Lee J, Lim KH, Weber A, Slankamenac K, Poon RT, Yang H, Ooi LL, Toh HC, Heikenwalder M (2012). Chemokine-driven lymphocyte infiltration: an early intratumoural event determining long-term survival in resectable hepatocellular carcinoma. Gut.

[22] Cheung ST, Leung KL, Ip YC, Chen X, Fong DY, Ng IO, Fan ST, So S (2005). Claudin-10 expression level is associated with recurrence of primary hepatocellular carcinoma. Clin Cancer Res.

[23] Chen KF, Chen HL, Tai WT, Feng WC, Hsu CH, Chen PJ, Cheng AL (2011). Activation of phosphatidylinositol 3-kinase/Akt signaling pathway mediates acquired resistance to sorafenib in hepatocellular carcinoma cells. J Pharmacol Exp Ther.

[24] Lin H, Dai T, Xiong H, Zhao X, Chen X, Yu C, Li J, Wang X, Song L (2010). Unregulated miR-96 induces cell proliferation in human breast cancer by downregulating transcriptional factor FOXO3a. PloS one.

[25] Pineau P, Volinia S, McJunkin K, Marchio A, Battiston C, Terris B, Mazzaferro V, Lowe SW, Croce CM, Dejean A (2010). miR-221 overexpression contributes to liver tumorigenesis. Proc Natl Acad Sci USA.

[26] Wang C, Ren R, Hu H, Tan C, Han M, Wang X, Zheng Y (2014). MiR-182 is up-regulated and targeting Cebpa in hepatocellular carcinoma. Chin J Cancer Res.

[27] Liu S, Guo W, Shi J, Li N, Yu X, Xue J, Fu X, Chu K, Lu C, Zhao J, Xie D, Wu M, Cheng S (2012). MicroRNA-135a contributes to the development of portal vein tumor thrombus by promoting metastasis in hepatocellular carcinoma. J Hepatol.

[28] Lim JY, Yoon SO, Seol SY, Hong SW, Kim JW, Choi SH, Lee JS, Cho JY (2013). Overexpression of miR-196b and HOXA10 characterize a poor-prognosis gastric cancer subtype. World J Gastroenterol.

[29] Coussens LM, Werb Z (2002). Inflammation and cancer. Nature.

[30] Sacconi A, Biagioni F, Canu V, Mori F, Di Benedetto A, Lorenzon L, Ercolani C, Di Agostino S, Cambria AM, Germoni S, Grasso G, Blandino R, Panebianco V (2012). miR-204 targets Bcl-2 expression and enhances responsiveness of gastric cancer. Cell Death Dis.

[31] Qiu T, Zhou X, Wang J, Du Y, Xu J, Huang Z, Zhu W, Shu Y, Liu P (2014). MiR-145, miR-133a and miR-133b inhibit proliferation, migration, invasion and cell cycle progression via targeting transcription factor Sp1 in gastric cancer. FEBS Lett.

[32] Wang J, Zhang J, Wu J, Luo D, Su K, Shi W, Liu J, Tian Y, Wei L (2012). MicroRNA-610 inhibits the migration and invasion of gastric cancer cells by suppressing the expression of vasodilator-stimulated phosphoprotein. Eur J Cancer.

[33] Liu Y, Liu H, Luo X, Deng J, Pan Y, Liang H (2015). Overexpression of SMYD3 and matrix metalloproteinase-9 are associated with poor prognosis of patients with gastric cancer. Tumour Biol.

[34] Yoon C, Cho SJ, Aksoy BA, Park DJ, Schultz N, Ryeom S, Yoon SS (2015). Chemotherapy resistance in diffuse type gastric adenocarcinoma is mediated by RhoA activation in cancer stem-like cells. Clin Cancer Res.

[35] Park JH, Lee BL, Yoon J, Kim J, Kim MA, Yang HK, Kim WH (2010). Focal adhesion kinase (FAK) gene amplification and its clinical implications in gastric cancer. Hum Pathol.

[36] Xiao SD, Meng XJ, Shi Y, Hu YB, Zhu SS, Wang CW (2002). Interventional study of high dose folic acid in gastric carcinogenesis in beagles. Gut.

[37] Hu J, La Vecchia C, Augustin LS, Negri E, de Groh M, Morrison H, Mery L (2013). Glycemic index, glycemic load and cancer risk. Ann Oncol.

[38] Lu C, Huang T, Chen W, Lu H (2015). GnRH participates in the self-renewal of A549-derived lung cancer stem-like cells through upregulation of the JNK signaling pathway. Oncol Rep.

[39] Cesen-Cummings K, Fernstrom MJ, Malkinson AM, Ruch RJ (1998). Frequent reduction of gap junctional intercellular communication and connexin43 expression in human and mouse lung carcinoma cells. Carcinogenesis.

[40] Pandiri AR, Sills RC, Ziglioli V, Ton TV, Hong HH, Lahousse SA, Gerrish KE, Auerbach SS, Shockley KR, Bushel PR, Peddada SD, Hoenerhoff MJ (2012). Differential transcriptomic analysis of spontaneous lung tumors in B6C3F1 mice: comparison to human non-small cell lung cancer. Toxicol Pathol.

[41] Scagliotti GV, Novello S (2012). The role of the insulin-like growth factor signaling pathway in non-small cell lung cancer and other solid tumors. Cancer Treat Rev.

[42] Raz G, Allen KE, Kingsley C, Cherni I, Arora S, Watanabe A, Lorenzo CD, Edwards VD, Sridhar S, Hostetter G, Weiss GJ (2012). Hedgehog signaling pathway molecules and ALDH1A1 expression in early-stage non-small cell lung cancer. Lung cancer (Amsterdam, Netherlands).

[43] Ji HF, Pang D, Fu SB, Jin Y, Yao L, Qi JP, Bai J (2013). Overexpression of focal adhesion kinase correlates with increased lymph node metastasis and poor prognosis in non-small-cell lung cancer. J Cancer Res Clin Oncol.

[44] Qiu T, Zhou L, Wang T, Xu J, Wang J, Chen W, Zhou X, Huang Z, Zhu W, Shu Y, Liu P (2013). miR-503 regulates the resistance of non-small cell lung cancer cells to cisplatin by targeting Bcl-2. Int J Mol Med.

[45] Kang J, Kim E, Kim W, Seong KM, Youn H, Kim JW, Kim J, Youn B (2013). Rhamnetin and cirsiliol induce radiosensitization and inhibition of epithelial-mesenchymal transition (EMT) by miR-34a-mediated suppression of Notch-1 expression in non-small cell lung cancer cell lines. J Biol Chem.

[46] Hu J, Cheng Y, Li Y, Jin Z, Pan Y, Liu G, Fu S, Zhang Y, Feng K, Feng Y (2014). microRNA-128 plays a critical role in human non-small cell lung cancer tumourigenesis, angiogenesis and lymphangiogenesis by directly targeting vascular endothelial growth factor-C. Eur J Cancer.

[47] Yanaihara N, Caplen N, Bowman E, Seike M, Kumamoto K, Yi M, Stephens RM, Okamoto A, Yokota J, Tanaka T, Calin GA, Liu CG, Croce CM (2006). Unique microRNA molecular profiles in lung cancer diagnosis and prognosis. Cancer cell.

[48] Avila MA, Berasain C, Torres L, Martin-Duce A, Corrales FJ, Yang H, Prieto J, Lu SC, Caballeria J, Rodes J, Mato JM (2000). Reduced mRNA abundance of the main enzymes involved in methionine metabolism in human liver cirrhosis and hepatocellular carcinoma. J Hepatol.

[49] Darpolor MM, Yen YF, Chua MS, Xing L, Clarke-Katzenberg RH, Shi W, Mayer D, Josan S, Hurd RE, Pfefferbaum A, Senadheera L, So S, Hofmann LV *In vivo* MRSI of hyperpolarized [1-(13)C]pyruvate metabolism in rat hepatocellular carcinoma. NMR Biomed.

[50] Kanehisa M, Goto S, Sato Y, Furumichi M, Tanabe M (2012). KEGG for integration and interpretation of large-scale molecular data sets. Nucleic Acids Res.

[51] Subramanian A, Tamayo P, Mootha VK, Mukherjee S, Ebert BL, Gillette MA, Paulovich A, Pomeroy SL, Golub TR, Lander ES, Mesirov JP (2005). Gene set enrichment analysis: a knowledge-based approach for interpreting genome-wide expression profiles. Proceedings of the National Academy of Sciences of the United States of America.

[52] Ma L, Huang Y, Zhu W, Zhou S, Zhou J, Zeng F, Liu X, Zhang Y, Yu J (2011). An integrated analysis of miRNA and mRNA expressions in non-small cell lung cancers. PloS one.

[53] Jang JS, Jeon HS, Sun Z, Aubry MC, Tang H, Park CH, Rakhshan F, Schultz DA, Kolbert CP, Lupu R, Park JY, Harris CC, Yang P (2012). Increased miR-708 expression in NSCLC and its association with poor survival in lung adenocarcinoma from never smokers. Clin Cancer Res.

[54] Benjamini Y, Hochberg Y (1995). Controlling the False Discovery Rate: A Practical and Powerful Approach to Multiple Testing. J R Stat Soc B (Methodological).

