# Supplementary material for: MiRSEA: Discovering the pathways regulated by dysfunctional MicroRNAs
Source: Oncotarget. 2016 Jul 26;7(34):55012–25. doi: 10.18632/oncotarget.10839 (PMC5342398; doi:10.18632/oncotarget.10839)
Supplement: Supplementary file 1 [file oncotarget-07-55012-s001.pdf]

MiRSEA: Discovering the pathways regulated by dysfunctional MicroRNAs

Supplementary Materials

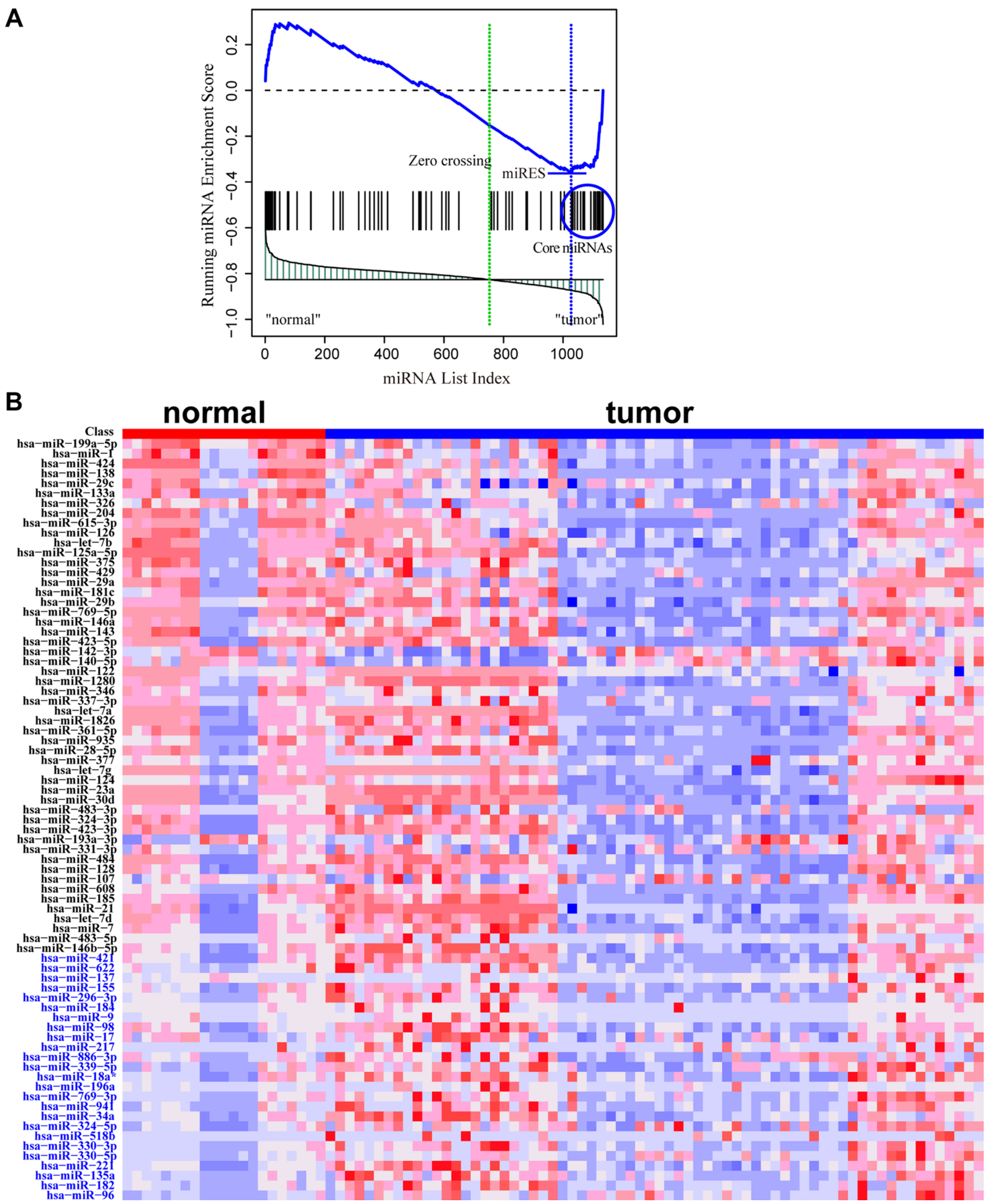

Supplementary Figure S1: Running enrichment score of the chemokine signaling pathway. (**A**) Running-sum statistic is calculated by walking down the miRNA list, and the maximum deviation from zero of the statistic is used as miRNA enrichment score (miRES) of the pathway. (**B**) Heatmap of the miRNAs in the pathway. Core miRNAs are marked with blue.

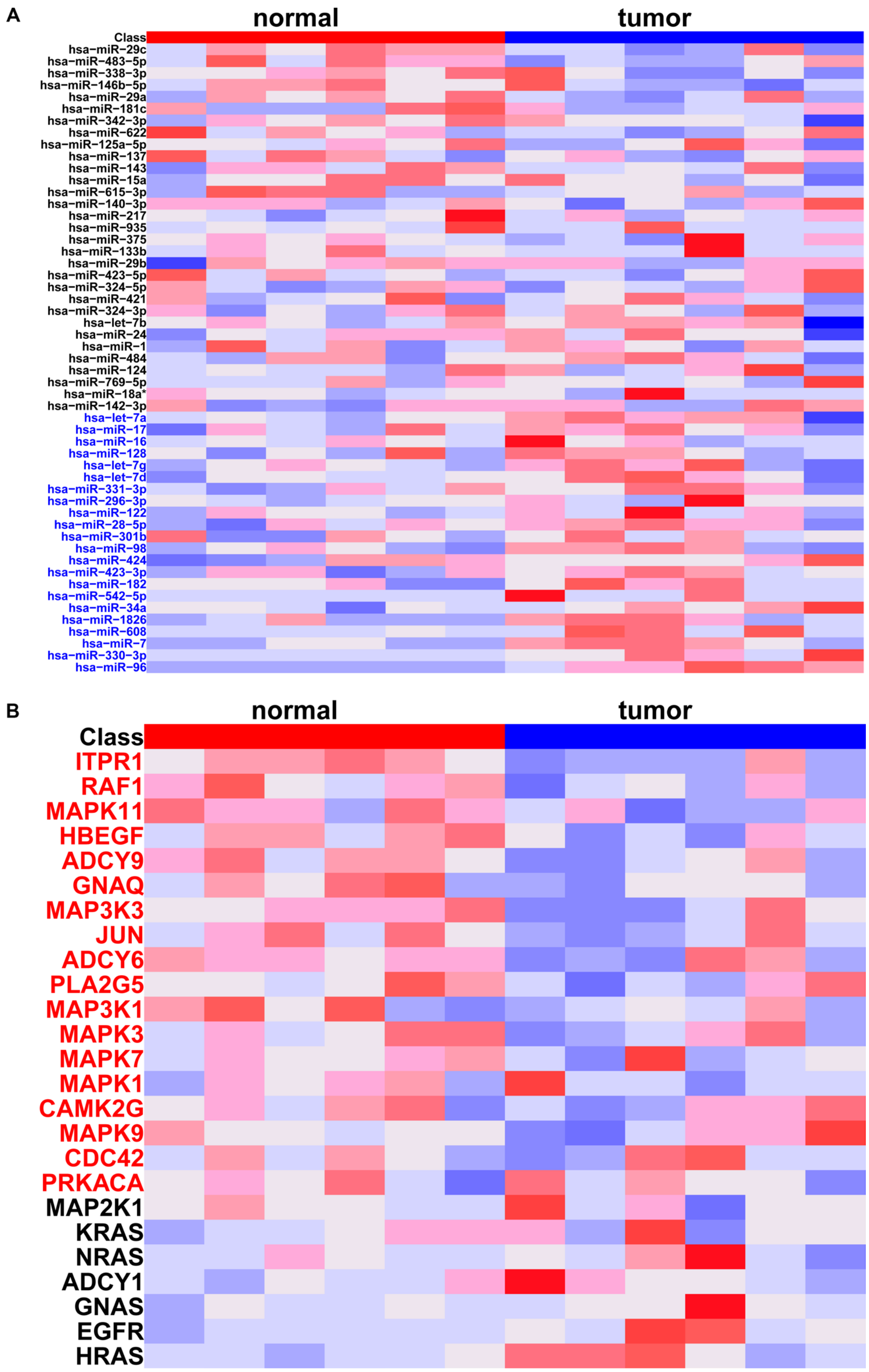

Supplementary Figure S2: (**A**) Heatmap of the miRNAs in the GnRH signaling pathway. Core miRNAs are marked with blue. (**B**) Heatmap of the mRNAs targeted by core miRNAs in the pathways. Down-regulated genes in the NSCLC samples are marked with red.

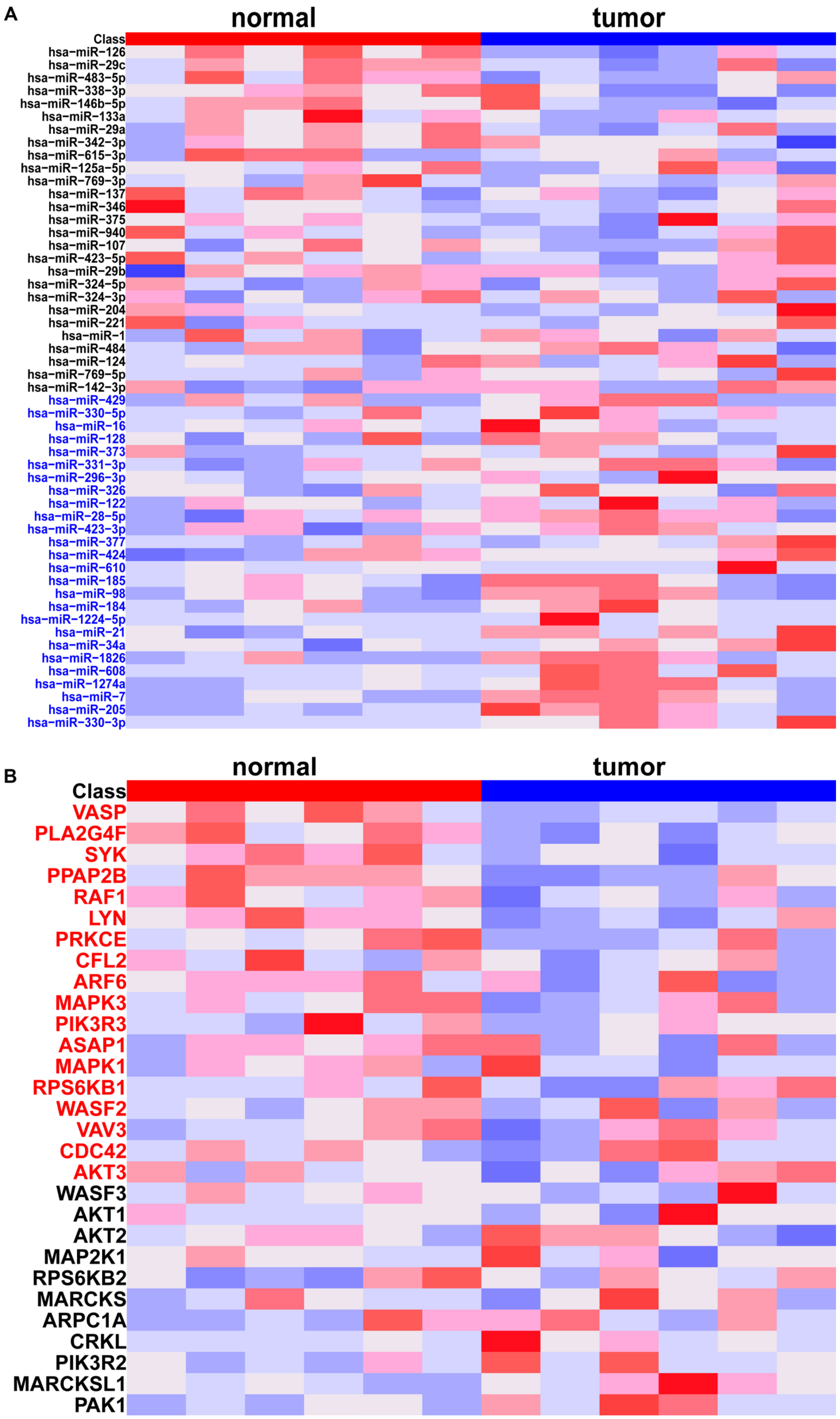

Supplementary Figure S3: (**A**) Heatmap of the miRNAs in the Fc gamma R-mediated phagocytosis pathway. Core miRNAs are marked with blue. (**B**) Heatmap of the mRNAs targeted by core miRNAs in the pathways. Down-regulated genes in the NSCLC samples are marked with red.

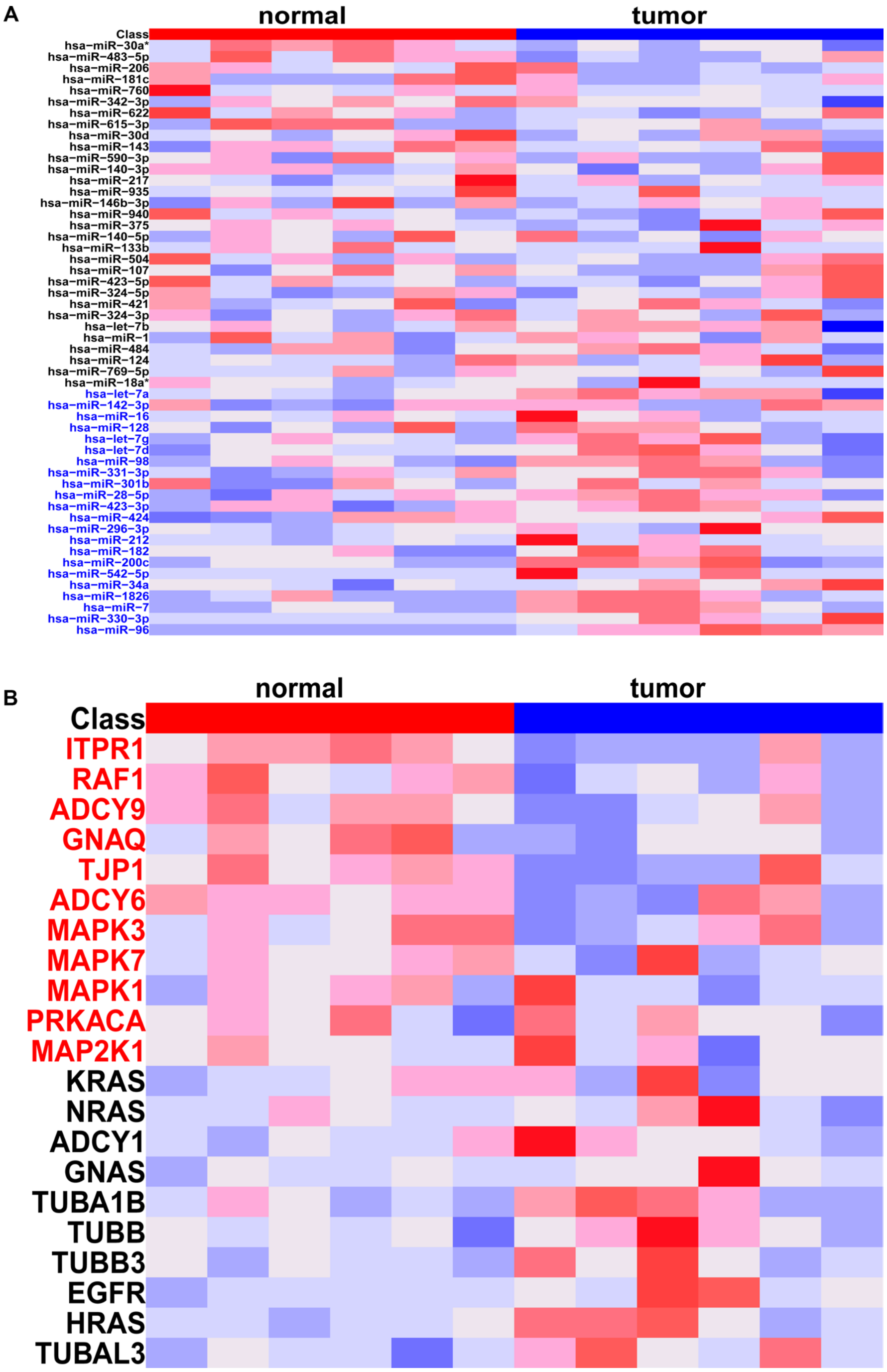

Supplementary Figure S4: (**A**) Heatmap of the miRNAs in the gap junction pathway. Core miRNAs are marked with blue. (**B**) Heatmap of the mRNAs targeted by core miRNAs in the pathways. Down-regulated genes in the NSCLC samples are marked with red.

Supplementary Table S1: Full list of ranked KEGG pathways identified by MiRSEA in the human hepatocellular carcinoma (HCC) dataset. See Supplementary_Table_S1.

Supplementary Table S2: Detail information for each miRNA in the sphingolipid metabolism pathway

#	miRNA	LIST LOC	miRSCORE	RES	CORE_ENRICHMENT
1	hsa-miR-1	4	0.898	0.223	YES
2	hsa-miR-125b	8	0.785	0.418	YES
3	hsa-miR-615-3p	9	0.781	0.615	YES
4	hsa-miR-375	20	0.643	0.768	YES
5	hsa-miR-769-5p	68	0.463	0.842	YES
6	hsa-miR-423-3p	757	−0.00093	0.23	NO
7	hsa-miR-331-3p	766	−0.0103	0.226	NO
8	hsa-miR-484	783	−0.0274	0.219	NO
9	hsa-miR-128	818	−0.0533	0.202	NO
10	hsa-miR-98	1065	−0.312	0.0614	NO

Supplementary Table S3: Detail information for each miRNA in the chemokine signaling pathway. See Supplementary_Table_S3.

Supplementary Table S4: Full list of ranked KEGG pathways identified by MiRSEA in the gastric adenocarcinoma dataset. See Supplementary_Table_S4.

Supplementary Table S5: Detail information for each miRNA in the leukocyte transendothelial migration pathway

#	miRNA	LIST LOC	miRSCORE	RES	CORE_ENRICHMENT
1	hsa-miR-204	1	1.24	0.122	YES
2	hsa-miR-133a	2	0.816	0.202	YES
3	hsa-miR-193a-3p	3	0.762	0.277	YES
4	hsa-miR-338-3p	4	0.676	0.343	YES
5	hsa-miR-610	5	0.65	0.407	YES
6	hsa-miR-137	6	0.534	0.459	YES
7	hsa-miR-339-5p	8	0.471	0.503	YES
8	hsa-miR-326	10	0.437	0.543	YES
9	hsa-miR-375	17	0.318	0.558	YES
10	hsa-miR-29c	20	0.298	0.582	YES
11	hsa-miR-142-3p	22	0.274	0.606	YES
12	hsa-miR-1280	23	0.265	0.632	YES
13	hsa-miR-886-3p	25	0.248	0.654	YES
14	hsa-miR-29b	42	0.162	0.626	NO
15	hsa-miR-423-5p	71	0.122	0.562	NO
16	hsa-miR-1	73	0.12	0.571	NO
17	hsa-miR-138	86	0.108	0.548	NO
18	hsa-miR-532-3p	102	0.0894	0.516	NO
19	hsa-miR-769-5p	122	0.0675	0.471	NO
20	hsa-miR-126	123	0.0631	0.477	NO
21	hsa-miR-146a	125	0.0627	0.481	NO
22	hsa-miR-331-3p	143	0.0456	0.439	NO
23	hsa-miR-324-5p	162	0.0272	0.392	NO
24	hsa-miR-29a	167	0.0214	0.383	NO
25	hsa-miR-615-3p	181	0.0135	0.349	NO
26	hsa-miR-30d	186	0.012	0.339	NO
27	hsa-miR-484	206	−0.00507	0.288	NO
28	hsa-miR-24	216	−0.0113	0.264	NO
29	hsa-miR-221	235	−0.0217	0.217	NO
30	hsa-miR-324-3p	248	−0.0306	0.188	NO
31	hsa-miR-342-3p	266	−0.0447	0.146	NO
32	hsa-miR-361-5p	309	−0.0893	0.0396	NO
33	hsa-miR-23a	327	−0.12	0.00494	NO
34	hsa-miR-455-3p	356	−0.166	−0.0553	NO
35	hsa-miR-128	357	−0.168	−0.0388	NO
36	hsa-miR-146b-5p	361	−0.18	−0.0293	NO
37	hsa-miR-155	388	−0.306	−0.0703	NO
38	hsa-miR-183	389	−0.307	−0.0401	NO
39	hsa-miR-1826	396	−0.359	−0.0212	NO
40	hsa-miR-98	403	−0.466	0.0082	NO

Supplementary Table S6: Detail information for each miRNA in the focal adhesion pathway.
See Supplementary_Table_S6.

Supplementary Table S7: Number of significant pathway identified by MiRSEA, Traditional method and Godard et al. method in human hepatocellular carcinoma, gastric adenocarcinoma, oral carcinoma, acute lymphoblastic leukemia and lung cancer datasets

Method<br>Dataset	MiRSEA		Traditional method		Godard et al. method	
	Up	Down	Up	Down	Up	Down
Human hepatocellular carcinoma	11	22	27	33	0	0
Gastric adenocarcinoma	0	15	29	41	0	48
Oral carcinoma	16	1	65	59	49	0
Acute lymphoblastic leukemia	35	12	8	1	0	0
Lung cancer	61	0	56	31	46	0

Supplementary Table S8: Pathways identified by three methods (MiRSEA, Traditional method, Godard et al. method) with FDR < 0.01 in the prostate cancer dataset. See Supplementary_Table_S8.
