# Supplementary material for: MiRSEA: Discovering the pathways regulated by dysfunctional MicroRNAs
Source: Oncotarget. 2016 Jul 26;7(34):55012–25. doi: 10.18632/oncotarget.10839 (PMC5342398; doi:10.18632/oncotarget.10839)
Supplement: Supplementary file 6 [file oncotarget-07-55012-s006.doc]

**Table S8. Pathways identified by three methods (MiRSEA, Traditional method, Godard et al. method) with FDR <0.01 in the prostate cancer dataset.**

| **Pathway** | **MiRSEA** | **Traditional method** | **Godard et al. method** |
| --- | --- | --- | --- |
| Sphingolipid metabolism | √ |  |  |
| Calcium signaling pathway | √ |  |  |
| Cell adhesion molecules (CAMs) | √ |  |  |
| Glycerolipid metabolism | √ |  |  |
| Non-homologous end-joining | √ |  |  |
| Drug metabolism - other enzymes | √ |  |  |
| Prion diseases | √ |  |  |
| Cysteine and methionine metabolism | √ |  |  |
| Focal adhesion | √ | √ |  |
| Adherens junction | √ | √ |  |
| Pyruvate metabolism | √ |  |  |
| MAPK signaling pathway | √ |  |  |
| Bladder cancer | √ | √ |  |
| Pathways in cancer | √ | √ |  |
| Starch and sucrose metabolism | √ |  |  |
| Type II diabetes mellitus | √ |  |  |
| Glioma | √ | √ |  |
| Endometrial cancer | √ |  |  |
| p53 signaling pathway | √ | √ |  |
| Prostate cancer | √ | √ |  |
| Viral myocarditis | √ |  |  |
| Colorectal cancer | √ | √ |  |
| Ribosome | √ | √ |  |
| Chemokine signaling pathway | √ | √ |  |
| Huntingtons disease | √ | √ |  |
| Tight junction | √ | √ |  |
| Melanogenesis | √ |  |  |
| Phosphatidylinositol signaling system | √ |  |  |
| Leishmania infection | √ |  |  |
| Renal cell carcinoma | √ |  |  |
| Antigen processing and presentation | √ |  |  |
| Fructose and mannose metabolism | √ |  |  |
| Pentose phosphate pathway | √ |  |  |
| Arrhythmogenic right ventricular cardiomyopathy |  | √ |  |
| Acute myeloid leukemia |  | √ |  |
| Cell cycle |  | √ |  |
| Chronic myeloid leukemia |  | √ |  |
| Dilated cardiomyopathy |  | √ |  |
| DNA replication |  | √ |  |
| Dorso ventral axis formation |  | √ |  |
| ECM receptor interaction |  | √ |  |
| Endometrial cancer |  | √ |  |
| [Epithelial Cell Signaling in Helicobacter pylori Infection](http://www.so.com/link?url=http%3A%2F%2Fwww.researchgate.net%2Fpublication%2F228868362_Epithelial_cell_signaling_in_Helicobacter_pylori_infection&q=EPITHELIAL+CELL+SIGNALING+IN+HELICOBACTER+PYLORI+INFECTION&ts=1457161921&t=3680b184c42addde38cdd9dabd8523d&src=haosou) |  | √ |  |
| ErbB signaling pathway |  | √ |  |
| Gap junction |  | √ |  |
| Glycolysis and gluconeogenesis |  | √ |  |
| Hypertrophic Cardiomyopathy - HCM |  | √ |  |
| Insulin signaling pathway |  | √ |  |
| [Leukocyte Transendothelial Migration](http://www.so.com/link?url=http%3A%2F%2Fwww.39kf.com%2Fcooperate%2Fqk%2Farteriosclerosis%2F0405%2F2007-05-18-365653.shtml&q=LEUKOCYTE+TRANSENDOTHELIAL+MIGRATION&ts=1457162036&t=8e808984b06022159d578aee0b19ca8&src=haosou) |  | √ |  |
| Melanoma |  | √ |  |
| Mismatch repair |  | √ |  |
| [Neurotrophin signaling pathway](http://www.so.com/link?url=http%3A%2F%2Fwww.genome.jp%2Fkegg%2Fpathway%2Fhsa%2Fhsa04722.html&q=NEUROTROPHIN+SIGNALING+PATHWAY&ts=1457162086&t=3672b18bf212d7a22117dc7f2e65d56&src=haosou) |  | √ |  |
| Non small cell lung cancer |  | √ |  |
| [Notch signaling pathway](https://en.wikipedia.org/wiki/Notch_signaling_pathway) |  | √ |  |
| Pancreatic cancer |  | √ |  |
| [Pathogenic Escherichia coli infection](http://www.so.com/link?url=http%3A%2F%2Fwww.wikipathways.org%2Findex.php%2FPathway%3AWP2272&q=PATHOGENIC+ESCHERICHIA+COLI+INFECTION&ts=1457162178&t=9c4484285c81ce7b75de219d278c9e0&src=haosou) |  | √ |  |
| Proteasome |  | √ |  |
| Pyrimidine metabolism |  | √ |  |
| [Regulation of actin cytoskeleton](http://www.so.com/link?url=http%3A%2F%2Fwww.genome.jp%2Fkegg%2Fpathway%2Fhsa%2Fhsa04810.html&q=REGULATION+OF+ACTIN+CYTOSKELETON&ts=1457162252&t=237c7bd66c2c7de8ac96e27ba48d0ef&src=haosou) |  | √ |  |
| Small cell lung cancer |  | √ |  |
| Spliceosome |  | √ |  |
| Ubiquitin-mediated proteolysis |  | √ |  |
| [Vibrio cholerae Infection](http://www.so.com/link?url=http%3A%2F%2Fwww.biomedsearch.com%2Fsci%2FVibrio-cholerae-Infection-Drosophilamelanogaster-Mimics%2F0000559870.html&q=VIBRIO+CHOLERAE+INFECTION&ts=1457162330&t=16637ae1a731fb04b97466c31267930&src=haosou) |  | √ |  |

# Pathways which were exclusively identified by MiRSEA were mark with red.
